# The role of Effortful Control and the Dual Pathway Model in Childhood Obesity

**DOI:** 10.1192/j.eurpsy.2022.172

**Published:** 2022-09-01

**Authors:** L. Vervoort, T. Naets

**Affiliations:** 1 Ghent University, Clinical Developmental Psychology, Ghent, Belgium; 2 Radboud University, Development Psychology, Nijmegen, Netherlands; 3 Radboud University, Behavioral Science Institute, Nijmegen, Netherlands; 4 Odisee University College, Department Health Care (dietetics),, Ghent, Belgium

**Keywords:** dual pathway of obesity, demonstration of intervention techniques, Effortful Control: top down regulation and bottom up reactivity, children and adolescents

## Abstract

Apart from other well-known psychological determinants of obesity, deficits in neuropsychological processes related to effortful control can be relevant predictors for weight problems and difficulties in weight loss. Deficits in top-down inhibition as well as in other processes such as strong bottom-up reactivity, like external eating, hinder resisting food temptations in an obesogenic environment, thus determining the risk for the development of overweight and obesity. Furthermore, these deficits are associated with less positive outcomes in weight loss interventions for subgroups of children and adolescents with severe obesity. Targeting those effortful control mechanisms underlying childhood obesity via cognitive behavioral techniques can facilitate behavioural change necessary to induce sustainable weight loss and weight control. In this presentation, I will present data on effortful control collected in a large sample of youngsters (n=572 participants, 51% boys, aged 7–19) with moderate to severe obesity. Results suggest that top-down inhibition and bottom-up external eating play a complex role in weight problems in certain subgroups of youngsters with obesity, stressing the importance of identifying subgroups for tailoring interventions. I will demonstrate a few science-based intervention techniques that have the potential to ameliorate effortful control capacities, and thus optimize treatment outcomes for those youngsters low in effortful control.

**Disclosure:**

No significant relationships.

